# Development and validation of portable, field-deployable Ebola virus point-of-encounter diagnostic assay for wildlife surveillance

**DOI:** 10.1186/s42522-021-00041-y

**Published:** 2021-05-24

**Authors:** Dania M. Figueroa, Eeva Kuisma, M. Jeremiah Matson, Alain U. Ondzie, Trent Bushmaker, Stephanie N. Seifert, Francine Ntoumi, Beatriz Escudero-Pérez, César Muñoz-Fontela, Chris Walzer, Sarah H. Olson, Cynthia Goma-Nkoua, Jean-Vivien Mombouli, Robert J. Fischer, Vincent J. Munster

**Affiliations:** 1 Laboratory of Virology, Division of Intramural Research, National Institute of Allergy and Infectious Disease, National Institutes of Health, Hamilton, MT USA; 2 Wildflife Conservation Society, Health Program, Bronx, NY USA; 3 Marshall University, Joan C. Edwards School of Medicine, Huntington, WV USA; 4 Fondation Congolaise pour la Recherche Médicale (FCRM), Brazzaville, Republic of Congo; 5 Bernhard Nocht Institute for Tropical Medicine, Bernhard Nocht Strasse 74, 20359 Hamburg, Germany; 6 German Center for Infection Research (DZIF), Partner Site Hamburg, Bernhard Nocht Strasse 74, 20359 Hamburg, Germany; 7 Department of Interdisciplinary Life Sciences, Research Institute of Wildlife Ecology, University of Veterinary Medicine, Vienna, Austria; 8 Laboratoire National de Santé Publique, Brazzaville, Republic of the Congo; 9 Rocky Mountain Laboratories, NIAID/NIH, 903S 4th Street, Hamilton, MT 59840 USA

**Keywords:** Ebola virus, Portable diagnostics, Wildlife surveillance, RT-qPCR, Zoonoses

## Abstract

**Abstract:**

Early detection of Ebola virus spillover into wildlife is crucial for rapid response. We developed and validated a portable, cold-chain independent Ebola virus RT-qPCR assay.

**Methods:**

The field syringe-based RNA extraction method was compared with a conventional laboratory-based spin-column RNA extraction method. Next, the qPCR efficiency and limit of detection of the assay was compared to standard laboratory-based reagents and equipment. The specificity of the assay was confirmed by testing against multiple Zaire Ebolavirus (EBOV) variants and other *ebolavirus* species. Lastly, swabs from an EBOV-infected non-human primate carcass, stored at environmental conditions mimicking central and west Africa, were analyzed to mimic in field conditions.

**Results:**

The syringe-based RNA extraction method performed comparably to a standard laboratory spin-column-based method. The developed assay was comparable in sensitivity and specificity to standard laboratory-based diagnostic assays. The assay specifically detected EBOV and not any of the other tested *ebolavirus* species, including Reston ebolavirus, Sudan ebolavirus, Bundibugyo ebolavirus, and Tai Forrest ebolavirus. Notably, the assays limit of detection for EBOV isolates were all below 4 genome copies/μL. The assay was able to detect EBOV in oral, nasal, thoracic cavity, and conjunctiva swabs obtained from an infected non-human primate.

**Conclusion:**

We developed a field-based Ebolavirus assay which is comparable in sensitivity and specificity to laboratory-based assays. Currently, the assay is being incorporated into wildlife carcass surveillance in the Republic of the Congo and is being adapted for other infectious disease agents.

## Introduction

Since its discovery in 1976, there have been 17 recorded Ebola virus (EBOV, species *Zaire ebolavirus*) outbreaks affecting humans in west and central Africa [1]. EBOV can cause mortality events in several wildlife species including gorillas, chimpanzees, and duikers [2, 3]. EBOV has been linked to severe population declines in the critically endangered western lowland gorilla (*Gorilla gorilla gorilla*) [4–6]. Multiple EBOV zoonotic spillover events during the 2001–2003 Gabon and Republic of Congo (RoC) outbreaks resulted from direct contact with infected gorilla, chimpanzee, and duiker carcasses [2]. Therefore, the ability to rapidly detect EBOV in wildlife carcasses has the potential to limit human contact with EBOV and prevent large-scale outbreaks. In addition, early detection of mortality events due to EBOV in wildlife could be used to limit further spread and aid in the design of data-driven countermeasures (e.g. wildlife vaccination) [7].

Rapidly diagnosing wildlife EBOV mortality events is hampered by a lack of diagnostic infrastructure in areas at high risk for outbreaks [8, 9]. Previously, we reported on the deployment of a mobile laboratory in Monrovia, Liberia, using Roche LightCycler 480 RNA Master Hydrolysis Probes (LC480) (Roche, Indianapolis IN) paired with a SmartCycler RT-qPCR instrument (Cepheid, USA) during the west Africa Ebola virus disease outbreak [10]. Although this setup is highly portable, relatively conventional laboratory infrastructure is still required for sample handling, RNA extraction, and RT-qPCR. Surveillance for EBOV in wildlife carcasses, particularly for high-risk species, would ideally be performed in remote locations at the site of the wildlife carcass, independent of a cold-chain, electric infrastructure or standard laboratory equipment.

Here we report on the development of an ultra-portable (3.6 kg) point-of-encounter (PoE) assay to perform EBOV wildlife mortality surveillance at the site of the carcass. The EBOV RT-qPCR assay uses the Biomeme Franklin Three9 real-time PCR thermocycler system (BM) (Biomeme, USA), comprised of a syringe-based extraction kit, RT-qPCR assay with thermostable reagents and a highly portable, 9-well, 3-channel, RT-qPCR instrument with a 6-h battery life.

## Methods

### RNA extraction

Virus was inactivated and RNA extracted following approved institutional protocols. Briefly, 140 μL of sample was added to 560 mL of AVL (Qiagen) and incubated at room temperature (RT) for 10 min; this mixture was then added to 560 μL of absolute ethanol. RNA was extracted using a QIAamp Viral RNA Mini Kit (Qiagen) according to the manufacturer’s instructions. The Biomeme M1 Sample Prep kit (M1) (Biomeme, USA) employs a 5-min syringe-based RNA extraction protocol and requires no electricity, cold-chain, or existing specialized laboratory equipment. Briefly, a sample preparation column is applied to a syringe and the sample is then pulled into the column using the syringe followed by a series of three wash and rinse buffers. The sample is then dried on the column before elution in 500 μL of elution buffer. The BM extraction was modified to include AVL and ethanol (Qiagen, USA) inactivation to ensure complete inactivation of EBOV [11].

### Real-time quantitative reverse-transcriptase PCR analysis

The RT-qPCRs in this manuscript were performed using either the SmartCycler (Cepheid, USA) or the Biomeme Franklin Three9 real-time PCR thermocycler system (Biomeme, USA) using LC480 reagents (Roche, USA) or BM lyophilized reagents. Both assays used the same primers and probes targeting the EBOV L gene: forward primer CAGCCAGCAATTTCTTCCAT, reverse primer TTTTCGGTTGCTGTTTCTGTG, and two probes FAM-ATCATTGGC/ZEN/RTACTGGAGGAGCAG-3IABkFQ/ and FAM-TCATTGGCG/ZEN/TACTGGAGGAGCAGG-3IABkFQ. This assay was designed to detect both West African and Central African EBOV lineages [10], Performance of reagents and primer-probe combinations were assessed between the LC480 reagents and the RealStar Ebola virus RT-PCR kit (RealStar) (Altona Diagnostics, Germany) on the BM instrument. The RealStar kit was selected because of its World Health Organization emergency use approval for EBOV diagnostics. The RealStar kit also targets the L gene (primer and probe sequences are proprietary). For all RT-qPCR reactions 5 μl of RNA sample was used in a total of 25 μl reaction volume. Exact genome copies/mL of each serially diluted sample was determined by Droplet Digital Polymerase Chain Reaction (ddPCR; Hercules, California, USA). All RT-qPCR tests were conducted in triplicate. The limit of detection (LoD) was determined as the genome copies/uL at which no virus was detected in any of a sample’s replicates.

### Viruses

BSL4 protocols, sample inactivation protocols, and standard operating procedures for removal of specimens from high containment were approved by the Institutional Biosafety Committee (IBC). EBOV viruses: EBOV/H.sapiens-tc/COD/1976/Mayinga-76 (EBOV/May), EBOV/H.sapiens-tc/GAB/1994/Gabon (EBOV/Gab) and EBOV/H.sapiens-tc/GUI/2014/Makona-C07 (EBOV/Mak)), and filovirus species: Sudan virus (, Bundibugyo virus, Tai Forest virus, Reston virus and Marburg virus were used in this manuscript.

### Postmortem sampling of EBOV-infected non-human primate

We studied a cynomolgus macaque included as control in EBOV vaccine studies and euthanized because of signs of Ebola Virus Disease and viremia. The animal was infected with EBOV/H.sapiens-tc/COD/1976/Mayinga-76. The animal carcass was maintained for 7 days at 27 °C and 60% relative humidity in environmental chamber (Caron, USA) to model the environmental conditions observed in West and Central Africa. Oral, nasal, thoracic cavity, and conjunctival swab samples were obtained at 0, 4-, and 7-days post-euthanasia [11]. All samples were extracted with the M1 kit and tested on the BM platform and the laboratory-based system.

### Statistical analyses

All statistical analyses were performed with GraphPad Prism Ver. 7.0 (GraphPad Software, San Diego, CA).

## Results

**Fig. 1:**
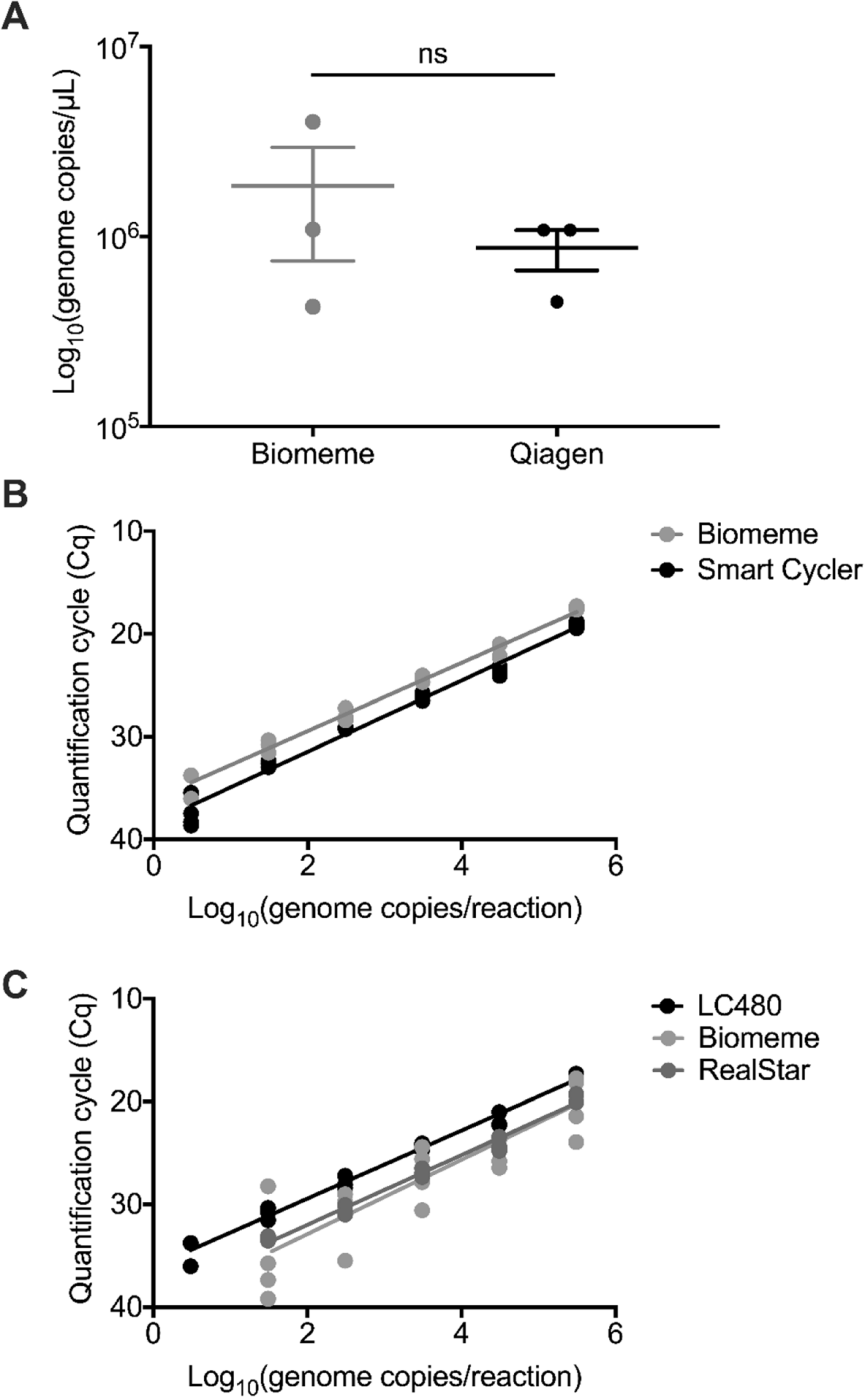
Validation of the PoE platform and assay. **a** Comparison of the efficiency of extraction of Ebola virus stocks H.sapiens-tc/COD/1995/Kikwit-15476 (EBOV/Kik) between the M1 Sample Prep or the Qiagen Viral RNA kit. The amount of RNA extracted by the two methods was not statistically different (Mann-Whitney test *p* > 0.05). Displayed are the three individual replicates for each extraction including mean and standard error of the mean. **b** Comparison of the Biomeme Franklin Three9 Real-Time PCR Thermocycler (BM) and the Smart Cycler using a ten-fold dilution series of EBOV/Kik, extracted using the Biomeme M1 Sample Prep, and measured using Roche LightCycler 480 RNA Master Hydrolysis Probes. **c** Comparison of the Biomeme lyophilized reagent system with the Roche LightCycler 480 RNA Master Hydrolysis Probes reagents and the Altona RealStar Ebola virus RT-PCR kit. A ten-fold dilution series was analyzed using the three reagent systems on the BM. The results of each individual replicate are depicted in the graphs. Genome copies/μL were calculated using a linear regression model derived from a tenfold dilution series analyzed with Droplet Digital PCR

We compared the sample RNA extraction efficiency between the Biomeme M1 Sample Prep kit and the QIAamp Viral RNA Mini Kit. No significant difference was observed between the Qiagen RNA extraction (1.826 × 10^7^ ± 4.968 × 10^8^ genome copies/mL, Mean ± SEM) and the BM AVL adapted extraction (8.097 × 10^8^ ± 2.558 × 10^8^ genome copies/mL Mean ± SEM) after adjusting for the different elution buffer volumes (Qiagen 60uL and Biomeme 500uL; Mann-Whitney, *p* > 0.05; Fig. 1a).

The qPCR efficiency and limit of detection (LoD) between instruments was evaluated by testing the LC480 reagents on both the BM and the SmartCycler (Fig. 1b). The qPCR efficiencies did not differ significantly at 96.26 and 105.68% for the SmartCycler and BM, respectively (ANCOVA, *p* > 0.05).

Next, the efficiency of the BM lyophilized reagents was compared to the LC480 reagents and the RealStar Ebola virus RT-PCR kit on the BM instrument (Fig. 1c). The amplification efficiency of the LC480 reagents on the BM instrument was 105.68%, efficiency of BM reagents was 107.42% and efficiency of the RealStar reagents was 105.82%. No significant difference between the amplification efficiencies were observed for any of the reagents (one-way ANOVA, *p* > 0.05).

The sensitivity and efficiency of the PoE assay was determined for EBOV strains from different outbreaks and phylogenetic lineages (Table 1). No significant difference was found between the amplification efficiencies of the analyzed EBOV strains (one-way ANOVA, *p* > 0.05). The sensitivity of the assay varied little between the strains, with the theoretical LoD calculated with Droplet Digital PCR (Bio-Rad, USA) and probit analysis ranging from 1.6 to 3.8 genome copies (Table 1). The specificity of the assay for EBOV was evaluated against other ebolavirus species, and all non-EBOV samples were negative with the EBOV assay but positive with their own respective primer and probe sets (primer and probe sequences available upon request). These results indicate that the developed assay is both sensitive and specific for all tested variants of EBOV.

**Table 1:** Ebola virus (*Zaire Ebolavirus*) strain comparison

EBOV strain	Amplification efficiency (%)	Limit of detection (genome copies)
EBOV/H.sapiens-tc/COD/1995/Kikwit-15476	107.42%	2.9
EBOV/H.sapiens-tc/GAB/1994/Gabon	97.83%	3.1
EBOV/H.sapiens-tc/COD/1976/Mayinga-76	90.15%	3.8
EBOV/H.sapiens-tc/GUI/2014/Makona-C07	99.05%	1.6

Ebola virus stocks were extracted with the Biomeme M1 Sample Prep. All samples were tested in triplicate. Amplification efficiencies were calculated using the straight-line portion of the regression curve. Limit of detection was calculated by absolute quantification with Droplet Digital PCR and probit analysis

**Fig. 2:**
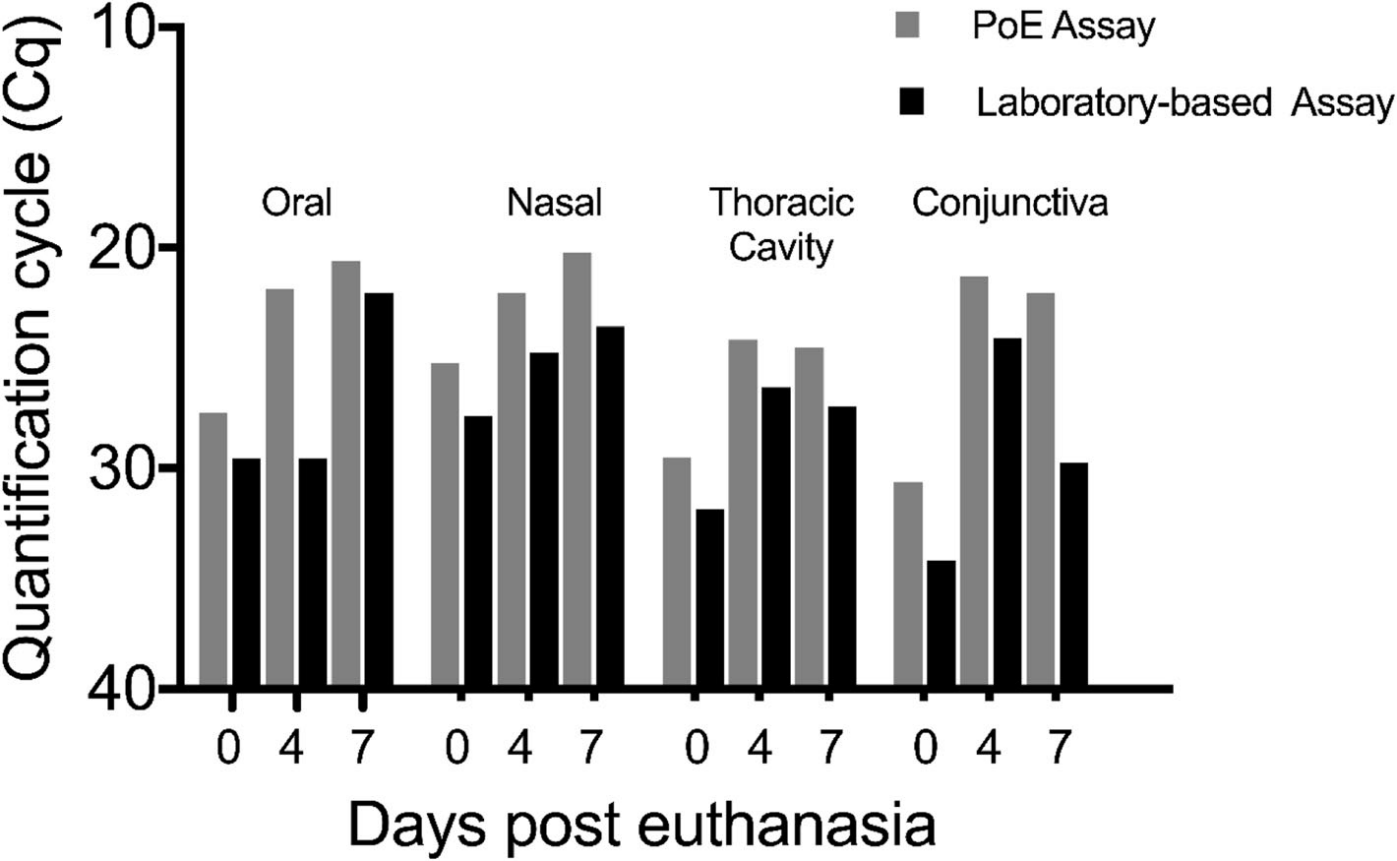
Analyses of carcass swabs from an EBOV-infected non-human primate. A non-human primate infected with H.sapiens-tc/GUI/2014/Makona-C07 (EBOV/Mak) was euthanized after reaching end-point criteria. After euthanasia, the carcass was stored at 27 °C and 60% relative humidity. Oral, nasal, thoracic cavity, and conjunctiva swabs were collected at 0-, 4-, and 7-days post euthanasia. All samples were extracted with the Biomeme M1 Sample Prep. C_q_ values were measured using the EBOV PoE assay and laboratory-based assay using the Roche LightCycler 480 RNA Master Hydrolysis Probes on the Smart Cycler

To evaluate the BM system on a realistic sample set, we obtained samples from an EBOV Makona-infected non-human primate carcass. After extraction, all samples were tested on the BM platform or the laboratory-based system. All samples were EBOV-positive on both assays (Fig. 2). The decrease in Ct values observed over time has been previously observed [12]. We hypothesis this is due to the breakdown of tissue and release of cellular viral RNA. Finally, long-term stability of the lyophilized PoE assay was determined. The reagents did not lose functionality after storage for over 2 years at ambient temperature (22 °C).

## Conclusion

**Fig. 3:**
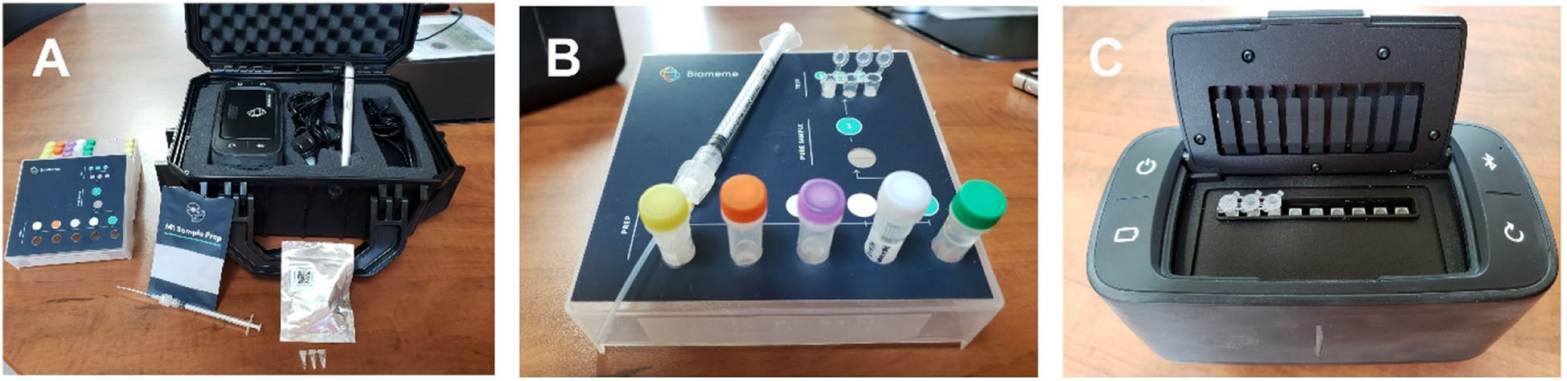
Overview of the Biomeme system. **a** complete set-up with PCR cycler and extraction kit. **b** Biomeme M1 syringe-based sample prep kit. **c** Biomeme battery operated mobile thermocycler

Several EBOV outbreaks have been associated with direct contact with infected wildlife carcasses. We have established a long-term wildlife-mortality-reporting network in RoC specifically designed for rapid EBOV detection [13]. Previously, our networks relied exclusively on sample analyses at the National Public Health laboratory in Brazzaville, RoC or via outside partners, and obtaining results could take anywhere from several days to months. To drastically reduce the turnaround time, we developed and validated an EBOV PoE assay. This PoE assay using the BM system was validated against the standard laboratory assay and RT-qPCR platforms and demonstrated comparable performance (Fig. 3). The assay had excellent specificity, and the LoD is well below the range of EBOV viral loads expected in an identifiable wildlife carcass [12, 14]. However, the data suggest that the variability of the PoE assay is greater as it reaches its LoD, indicating that this assay should not be used for quantification but rather as a preliminary diagnostic tool. The primary goal was to develop a PoE assay that could be incorporated into a previously-established safe sampling protocol and to perform rapid EBOV diagnostics in the field at the site of the wildlife carcass [13]. This assay will be deployed in the field in RoC to assess the EBOV infection status of encountered wildlife carcasses. Whereas the initial testing will be performed on-site, sample results will be confirmed at the National Public Health laboratory in Brazzaville. The inclusion of an EBOV PoE assay greatly shortens the response time of the early warning system in RoC, where both humans and great apes are at risk for infection with EBOV. Future studies are focused on adapting the PoE platform to include other high consequence wildlife infectious diseases, including those caused by *Bacillus* sp., *Yersinia pestis*, and monkeypox virus [15].

## Data Availability

NA.
